# Matching Clinical Diagnosis and Amyloid Biomarkers in Alzheimer’s Disease and Frontotemporal Dementia

**DOI:** 10.3390/jpm11010047

**Published:** 2021-01-14

**Authors:** Giulia Giacomucci, Salvatore Mazzeo, Silvia Bagnoli, Matteo Casini, Sonia Padiglioni, Cristina Polito, Valentina Berti, Juri Balestrini, Camilla Ferrari, Gemma Lombardi, Assunta Ingannato, Sandro Sorbi, Benedetta Nacmias, Valentina Bessi

**Affiliations:** 1 Department of Neuroscience, Psychology, Drug Research and Child Health, University of Florence (NEUROFARBA), Azienda Ospedaliera-Universitaria Careggi, Largo Brambilla 3, 50134 Florence, Italy; giuliagiacomucci.md@gmail.com (G.G.); salvatore.mazzeo@unifi.it (S.M.); silvia.bagnoli@unifi.it (S.B.); sonia_padiglioni@libero.it (S.P.); cristina.polito@unifi.it (C.P.); juri.balestrini@gmail.com (J.B.); camilla.ferrari@unifi.it (C.F.); ingannato.assunta@gmail.com (A.I.); sandro.sorbi@unifi.it (S.S.); benedetta.nacmias@unifi.it (B.N.); 2 IRCCS Fondazione Don Carlo Gnocchi, Via Scandicci 269, 50143 Florence, Italy; gemmalomb@gmail.com; 3 Faculty of Medicine and Surgery, University of Florence, Largo Brambilla 3, 50134 Florence, Italy; matteo.casini3@stud.unifi.it; 4 Department of Biomedical, Experimental and Clinical Sciences “Mario Serio”, University of Florence, Via Giovanni Battista Morgagni 50, 50134 Florence, Italy; valentina.berti@unifi.it; 5 Nuclear Medicine Unit, Azienda Ospedaliero-Universitaria Careggi, Largo Piero Palagi 1, 50139 Florence, Italy

**Keywords:** Alzheimer’s disease, frontotemporal dementia, CSF biomarkers, amyloid-PET

## Abstract

Background: The aims of this study were to compare the diagnostic accuracy, sensitivity, specificity, and positive and negative predictive values (PPV, NPV) of different cerebrospinal fluid (CSF) amyloid biomarkers and amyloid-Positron Emission Tomography (PET) in patients with a clinical diagnosis of Alzheimer’s disease (AD) and Frontotemporal Dementia (FTD); to compare concordance between biomarkers; and to provide an indication of their use and interpretation. Methods: We included 148 patients (95 AD and 53 FTD), who underwent clinical evaluation, neuropsychological assessment, and at least one amyloid biomarker (CSF analysis or amyloid-PET). Thirty-six patients underwent both analyses. One-hundred-thirteen patients underwent Apolipoprotein E (ApoE) genotyping. Results: Amyloid-PET presented higher diagnostic accuracy, sensitivity, and NPV than CSF Aβ_1–42_ but not Aβ_42/40_ ratio. Concordance between CSF biomarkers and amyloid-PET was higher in FTD patients compared to AD cases. None of the AD patients presented both negative Aβ biomarkers. Conclusions: CSF Aβ_42/40_ ratio significantly increased the diagnostic accuracy of CSF biomarkers. On the basis of our current and previous data, we suggest a flowchart to guide the use of biomarkers according to clinical suspicion: due to the high PPV of both amyloid-PET and CSF analysis including Aβ_42/40_, in cases of concordance between at least one biomarker and clinical diagnosis, performance of the other analysis could be avoided. A combination of both biomarkers should be performed to better characterize unclear cases. If the two amyloid biomarkers are both negative, an underlying AD pathology can most probably be excluded.

## 1. Introduction

Alzheimer’s disease (AD) is the most prevalent type of dementia and it has an increasing incidence worldwide [1]. AD progression has an impact on the length and quality of a patient’s life and represents a healthcare and economic burden. Therefore, it requires therapies able to stop disease progression [1].

Until 2007, AD diagnosis was based on clinical symptoms and cognitive examination, while a defined diagnosis was possible only by postmortem pathological confirmation [2]. The diagnosis of AD dementia has undergone a major change, from a purely clinical entity [2] toward a clinicobiological classification. In fact, in 2007 and then in 2014, the International Working Group (IWG) for New Research Criteria for the Diagnosis of Alzheimer’s Disease introduced a set of revised and updated criteria. They proposed to anchor the diagnosis of AD on the presence of biomarkers [3] whose practical application might allow earlier intervention in the prodromal stages of the disease and also facilitate diagnosis in doubtful cases.

The major biochemical biomarkers of AD pathology include: cerebrospinal fluid (CSF) 42 amino acid isoform of amyloid β (Aβ_1–42_) [4] and amyloid β 42/40 ratio (Aβ_42/40_) [5], which mirror the deposition of Aβ in brain tissue, and CSF total tau (t-tau) and hyperphosphorylated tau (p-tau), to assess neurofibrillary tangles and neuronal loss in the brain [6,7,8]. Amyloid-β pathology can also be detected in vivo by positron emission tomography (PET) using amyloid-β radiotracers such as [^11^C]Pittsburgh compound-B (PIB), [^18^F]Florbetapir, [^18^F]Florbetaben, or [^18^F]Flutemetamol, which allows one to directly visualize fibrillary amyloid-β deposits in brain tissue [9,10,11,12].

After the introduction of IWG criteria [3], biomarkers became the cornerstone of this new clinical-biological classification of AD, since they strongly improved the diagnostic accuracy, sensitivity, and specificity of previous clinical criteria. However, despite the high concordance with histopathology [8,9], there are some caveats in the validation of the use of biomarkers. PET and CSF have been included as equal alternatives to diagnostic criteria for both research [3,13,14] and clinical practice [15,16,17], although they measure amyloid in different pools (CSF and cortical brain tissue). However, several studies have shown that in 10–20% of patients, these modalities yield discordant results [18,19,20]. This discordance between amyloid biomarkers represents a challenging topic to explore.

The positivity of amyloid burden biomarkers has been found in other neurodegenerative diseases such as Frontotemporal Dementia (FTD). In fact, in rare cases, FTD patients might present a co-existing AD pathology [21]. Some cases of non-fluent and semantic variants of Primary Progressive Aphasia (nfv-PPA and sv-PPA) could be correlated to AD [22,23].

In an effort to improve the reliability of an AD diagnosis, it is important to distinguish between AD and other types of dementia that are not characterized by amyloid pathology, since the cognitive symptoms are often diffuse and overlap with other disorders [24,25].

In this study, we focused on a group of patients selected from a clinical setting cohort in order to provide an indication on the use of CSF biomarkers and amyloid-PET. In particular, the aims of this study were: (1) to compare the diagnostic accuracy, sensitivity, specificity, and positive and negative predictive values of different amyloid biomarkers in distinguishing between patients with clinical diagnosis of AD or FTD; (2) to compare the concordance between amyloid-PET and CSF biomarkers; and (3) to provide a flowchart to better clarify the use and interpretation of Aβ biomarkers according to clinical suspicion.

## 2. Materials and Methods

### 2.1. Patients

We included 148 patients who were referred to the Centre for Alzheimer’s Disease and Adult Cognitive Disorders of Careggi Hospital in Florence between January 2010 and March 2020 for evaluation of cognitive decline. All patients underwent a comprehensive family and clinical history, neurological examination, extensive neuropsychological investigation, estimation of premorbid intelligence, and assessment of depression. A positive family history was defined as one or more first-degree relatives with documented cognitive decline. All patients underwent measurement of at least one biomarker of amyloid burden: CSF biomarker analysis or amyloid-PET brain scan.

We included patients who met the following inclusion criteria: (1) patients who received a clinical diagnosis of AD according to the NIA-AA criteria, including the atypical variant [16], and (2) patients who received a clinical diagnosis of FTD according to the Neary criteria, including the behavioral variant (bv-FTD), non-fluent variant, and semantic variants of primary progressive aphasia (nfv-PPA and sv-PPA) [26].

Therefore, we finally included 95 AD and 53 FTD patients. Thirty-six patients (23 AD and 13 FTD) underwent both CSF biomarker analysis and amyloid-PET brain scan. Aβ pathology was defined as positive (Aβ+) if at least one of the amyloid biomarkers (CSF or amyloid PET) revealed the presence of Aβ pathology, or negative (Aβ-) if none of the biomarkers revealed the presence of Aβ pathology. We further classified patients as CSFAβ+ if they had at least one of Aβ_1–42_ or Aβ_42/40_ ratio positive, or CSF Aβ- if none of the CSF biomarkers revealed the presence of Aβ pathology.

One-hundred-thirteen subjects (72 AD and 41 FTD) underwent Apolipoprotein E (ApoE) genotyping: ApoE genotype was coded as ApoE ε4- (no ApoE ε4 alleles) and ApoE ε4+ (presence of one or two ApoE ε4 alleles).

Study procedures and data analysis were performed in accordance with the Declaration of Helsinki and with the ethical standards of the Committee on Human Experimentation of our Institute. The study was approved by the local Institutional Review Board (reference 15691oss). All individuals involved in this research agreed to participate and agreed to have details and results of the research about them published.

### 2.2. Neuropsychological Assessment

All subjects were evaluated by an extensive neuropsychological test battery standardized and described in further detail elsewhere [27]. The test battery consisted of global measurements (Mini-Mental State Examination), tasks exploring verbal and spatial short-term memory (Digit Span; Corsi Tapping Test), verbal long-term memory (Five Words and Paired Words Acquisition; Recall after 10 min; Recall after 24 h; Babcock Short Story Immediate and Delayed Recall), and language (Token Test; Category Fluency Task) [27]. Visuospatial abilities were also evaluated by Rey–Osterrieth Complex Figure copy and visuospatial long-term memory was assessed by means of recall of the Rey–Osterrieth Complex Figure test [28]. Attention/executive function was explored by means of Dual Task [29], the Phonemic Fluency Test [30], and the Trail Making Test [31]. Everyday memory was assessed by means of the Rivermead Behavioral Memory Test (RBMT) [32]. All raw test scores were adjusted for age, education, and gender according to the correction factor reported in validation studies for the Italian population [27,28,29,30,31,32]. Pre-morbid intelligence was estimated by the TIB (*Test di Intelligenza Breve*) [33], an Italian version of the National Adult Reading Test [34]. The presence and severity of depressive symptoms were evaluated by means of the 22-item Hamilton Depression Rating Scale (HRSD) [35].

### 2.3. PET Imaging Acquisition and Analysis

Fifty-eight patients (39 AD and 19 FTD) underwent amyloid-PET. Amyloid-PET imaging was performed according to standard national and international guidelines [36], with any of the available ^18^F-labeled tracers ([18]Florbetaben (FBB)—Bayer-Piramal; [18]Flutemetamol (FMM)—General Electric). Images were rated as either positive or negative according to the criteria defined by the manufacturers.

### 2.4. CSF Collection and Biomarkers Analysis

The CSF samples were collected by lumbar puncture, then immediately centrifuged and stored at −80 °C until performing the analysis. Two different methods of analysis were used to establish CSF measurements. In 59 patients (39 AD and 20 FTD), Aβ_1–42_, t-tau, and p-tau were measured with ELISA kits (INNOTEST Abeta _1–42_, INNOTEST PHOSPHO P Tau (181), and INNOTEST HTAU AG.). Cut-offs for normal values were: Aβ_1–42_ > 600 pg/mL; t-tau < 300 pg/mL; and p-tau < 60 pg/mL [37]. For 67 patients (40 AD and 27 FTD), Aβ_1–42_, Aβ_42/40_ ratio, t-tau, and p-tau were measured using a chemiluminescent enzyme immunoassay (CLEIA) analyzer LUMIPULSE G600 (Lumipulse Beta Amyloid_1–40_, Lumipulse Beta Amyloid_1–42_, Lumipulse GTotal Tau, and Lumipulse GPhospho Tau (181)). Cut-offs for normal values were: for Aβ_1–42_ > 670 pg/mL; Aβ_42/40_ ratio > 0.062; t-tau < 400 pg/mL; and p-tau < 60 pg/mL [38]. Reagent kits were obtained from Fujirebio.

### 2.5. Apolipoprotein E ε4 Genotyping, FAD and FTD Genes Mutation Analysis

A standard automated method (QIAcube, QIAGEN) was used to isolate DNA from peripheral blood samples. ApoE genotypes were investigated by high-resolution melting analysis (HRMA) [39]. Two sets of PCR primers were designed to amplify the regions encompassing rs7412 [NC_000019.9:g.45412079C>T] and rs429358 (NC_000019.9:g.45411941T>C). The samples with known ApoE genotypes, which had been validated by DNA sequencing, were used as standard references.

All the coding exons and the intron/exon boundaries of the familial AD genes (APP, PSEN1, and PSEN2) and frontotemporal dementia genes (GRN and MAPT) were amplified by polymerase chain reaction (PCR) using primers designed with Primer3 software (http://bioinfo.ut.ee/primer3-0.4.0/primer3/) [40]. The analysis was performed using high-resolution melting analysis (HRMA) followed by direct sequencing of amplicons showing heteroduplex (310 ABI PRISM Genetic Analyzer; Applied Biosystems, Foster City, USA). C9orf72 repeat expansion was searched using the repeat-primed PCR and automatic sequencing (3700 ABI PRISM Genetic Analyzer; Applied Biosystem, Foster City, USA); the characteristic stutter amplification pattern was considered as an indication of pathogenic repeat expansion (>22 repeats).

### 2.6. Statistical Analysis

Patient groups were characterized using means and standard deviations (SD). We tested for the normality distribution of the data using the Kolmogorov–Smirnov test. Depending on the distribution of our data, we used t-tests or non-parametric Mann–Whitney U tests for between-group comparisons and Pearson’s correlation coefficient or non-parametric Spearman’s ρ (rho) to evaluate correlations between groups’ numeric measures. We used a Chi-square test to compare categorical data and calculated the effect size by Cohen’s d for numeric measures and Cramer’s V for categorical data. All statistical analyses were performed with SPSS software v.25 (SPSS Inc., Chicago, IL, USA) and the computing environment R 4.0.3 (R Foundation for Statistical Computing, Vienna, 2013). The significance level was set at *p* < 0.05.

### 2.7. Data Availability

Raw data that support the findings of this study are not presented due to ethical and patient data security reasons but will be shared upon request from any qualified investigator.

## 3. Results

### 3.1. Description of the Sample 

There were no statistically significant differences between AD and FTD groups with respect to age at onset of symptoms, age at baseline evaluation, disease duration (time from onset of symptoms and baseline evaluation), age at CSF analysis, age at amyloid-PET, family history of dementia, sex, years of education, MMSE, and HDRS. Proportion of ApoE ε4+ was higher in the AD group compared to FTD (χ^2^ = 7.077 *p* = 0.008) (Table 1). In two nfv-PPA patients, the p.T272Sfs *10 and p.C149Lfs*10 GRN gene mutation were detected, described elsewhere [41,42]. We did not find any other variations in analyzed genes (PSEN1, PSEN2, APP, MAPT, and C9orf72).

### 3.2. Aβ Biomarkers Positivity Prevalence

Seventy-seven AD (81.05%) and 12 FTD (22.64%) patients were Aβ+. Aβ+ pathology proportion was higher in AD compared to FTD (χ^2^ = 48.42 *p* < 0.001).

Concerning CSF biomarkers, 48 AD (60.75%) and 8 FTD (17.02%) had positive Aβ_1–42_ (χ^2^ = 22.83 *p* < 0.001); 31 AD (75.60%) and 5 FTD (17.85%) had positive Aβ_42/40_ (χ^2^ = 22.24 *p* < 0.001). Fifty-one AD (64.55%) and 9 FTD (19.14%) were CSF Aβ+ (χ^2^ = 24.36 *p* < 0.001). Aβ_1–42_ and Aβ_42/40_ were both positive in 28 AD patients (70.00%) and in 4 FTD patients (14.81%).

Out of 58 patients who underwent amyloid-PET, 35 AD (89.74%) and 4 FTD subjects (21.05%) had positive scans for amyloid deposition (χ^2^ = 27.37 *p* < 0.001).

Considering the group of patients who underwent both CFS biomarkers analysis and amyloid-PET, 23 AD (100%) and 4 FTD (30.76%) were Aβ+ (χ^2^ = 21.23 *p* < 0.001).

### 3.3. Concordance between CSF and Amyloid PET

Considering the group of patients who underwent both CFS biomarkers analysis and amyloid-PET, we evaluated the concordance among the two Aβ biomarkers. Concordance was very low in AD patients, while it was significantly higher in the FTD group (39.13%, 95% C.I. 19.18–59.08 vs. 76.92%, 95% C.I. 54.02–99.83) (Table 2). CSF Aβ biomarkers and amyloid-PET were discordant in 17 patients (14 AD and 3 FTD). Of the 14 discordant patients, 13 had positive amyloid-PET but negative CSF Aβ biomarkers (CSF-/PET+). Two out of the three FTD discordant cases were CSF Aβ+ but had negative amyloid-PET (CSF+/PET-). One FTD patient presented both positive CSF Aβ biomarkers and positive amyloid-PET (CSF+/PET+). All FTD patients with at least one positive Aβ biomarker had a diagnosis of sv-PPA.

To assess if the discordance between CSF Aβ biomarkers and amyloid-PET was influenced by demographic features, we divided patients into two groups: patients whose biomarkers were both positive and patients with only one positive biomarker. There were no statistically significant differences between the two groups with respect to demographic features, MMSE, and ApoE ε4 prevalence. We also found no differences when we considered AD and FTD patients separately. Amyloid-PET was performed before CSF analysis in FTD patients (*p* = 0.034) (Table 1).

As regards the AD CSF-/PET+ patient subgroup, CSF Aβ_1–42_ was available for 10 cases: from a qualitative point of view, CSF Aβ_1–42_ levels were lower than 700 pg/mL in 9 cases, only slightly higher than the cut-off values. CSF Aβ_42/40_ ratio was available for three cases: similarly, two of them had CSF Aβ_42/40_ ratio values less than 0.7, slightly higher than the cut-off values. Moreover, they underwent CSF analysis more than 3 months before amyloid-PET. On the other hand, the only CSF+/PET- case underwent CSF analysis 2 months before amyloid-PET.

### 3.4. Concordance between Aβ_1–42_ and Aβ_42/40_

Concordance between Aβ_1–42_ and Aβ_42/40_ was 89.55% (95% C.I. 82.33–96.88) in the whole cohort, 92.25% (95% I.C. 84.34–100) in AD patients, and 85.19% (95% C.I. 71.75–98.59) in FTD patients (Table 2). In order to assess whether the discordance was influenced by demographic features, we divided patients into two groups: patients with positive Aβ_1–42_ and negative Aβ_42/40_, and patients with negative Aβ_1–42_ and positive Aβ_42/40_. There were no statistically significant differences between the two groups with respect to demographic features, MMSE, and ApoE ε4 prevalence, and also when we considered AD patients only.

From a qualitative point of view, all three AD discordant patients had positive Aβ_42/40_ but negative Aβ_1–42_.

### 3.5. Diagnostic Accuracy of Amyloid Burden Biomarkers

We evaluated the diagnostic accuracy of single or combined Aβ biomarkers. In the whole cohort, concordance between clinical diagnosis and Aβ pathology was 79.73% (95% C.I. 73.25–86.21). When we considered the subgroup of patients who underwent both CFS biomarkers analysis and amyloid-PET, the concordance was 88.89% (95% C.I. 78.62–99.16).

In order to assess if the discordance between amyloid biomarkers and clinical diagnosis was influenced by demographic features, we divided patients into two groups: the first including Aβ+ AD and Aβ- FTD (concordant cases), the second including Aβ- AD and Aβ+ FTD (discordant cases). There were no statistically significant differences between the two groups with respect to demographic features, MMSE, and ApoE ε4 prevalence. We also found no differences when we considered AD and FTD patients separately for this analysis. Considering Aβ+ FTD patients, we found a trend to significance for the ApoE ε4 allele (*p* = 0.065), which was more frequent in Aβ+ FTD patients.

**Figure 1:**
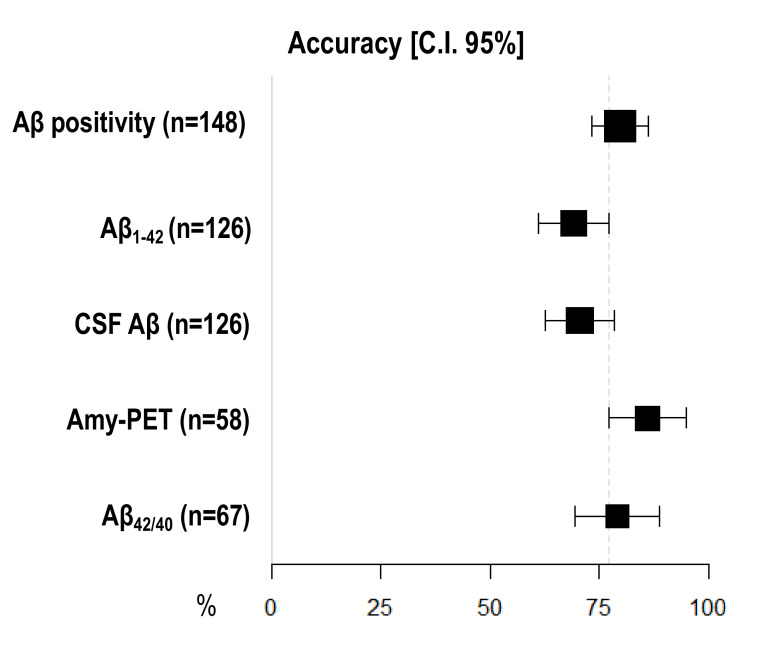
**Comparison of diagnostic accuracy among different Aβ biomarkers**. *X*-axis represents diagnostic accuracy in % (95% C.I.); different amyloid biomarkers are shown in *Y*-axis. The area of each square is proportional to the degree of the distribution of values of diagnostic accuracy of each biomarker and to the number of patients for whom each biomarker is available. The whiskers represent 95% C.I. The dotted vertical lines separate accuracy values that are significantly different.

Amyloid-PET showed the highest diagnostic accuracy, statistically significantly higher than CSF Aβ_1–42_ (86.21%, 95% I.C. 77.33–95.08 vs. 69.05, 95% C.I. 60.98–77.12), but not than CSF Aβ_42/40_ ratio (79.10%, 95% C.I. 69.37–88.84) and CSF Aβ (70.63%, 95% C.I. 62.68–78.59) (Figure 1) (Table 3).

**Figure 2:**
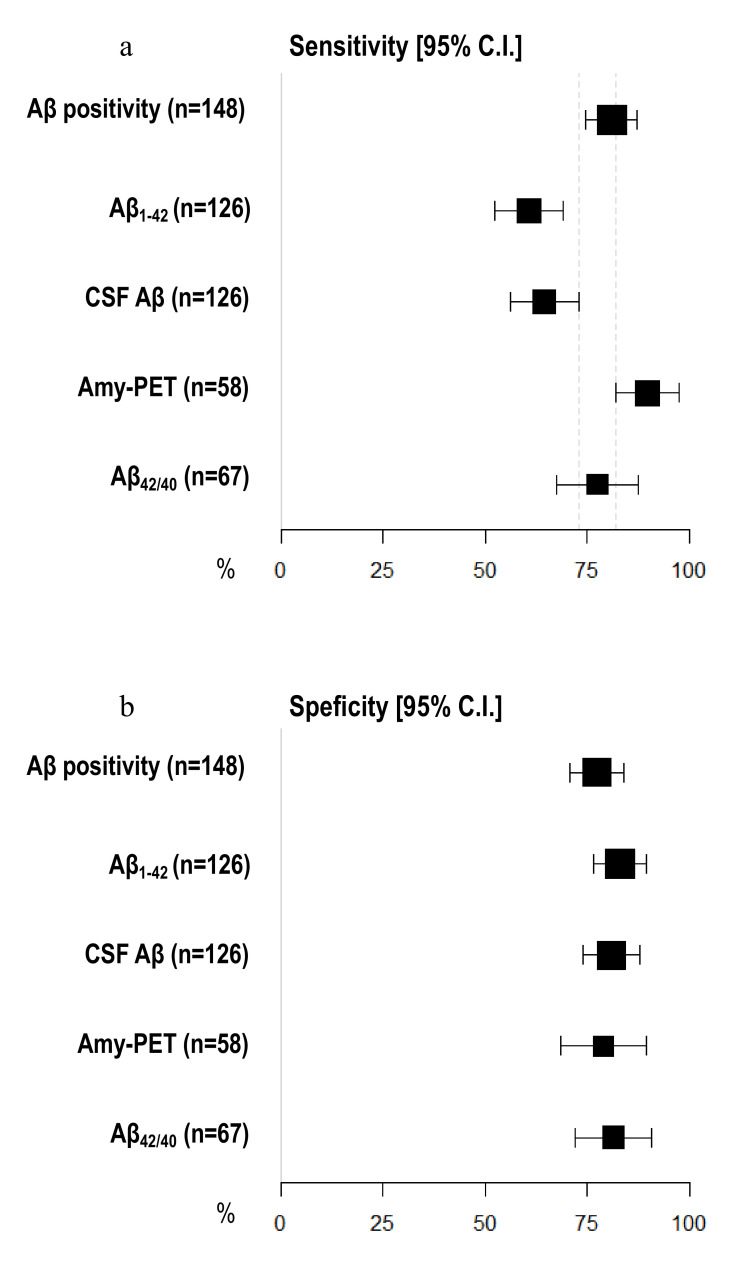
**Comparison of sensitivity and specificity among different Aβ biomarkers.** (**a**) *X*-axis represents sensitivity in % (95% C.I.); different amyloid biomarkers are shown in *Y*-axis. (**b**) *X*-axis represents specificity in % (95% C.I.); different amyloid biomarkers are shown in *Y*-axis. The area of each square is proportional to the degree of the distribution of values of sensitivity and specificity of each biomarker and to the number of patients for whom each biomarker is available. The whiskers represent 95% C.I. The dotted vertical lines separate sensitivity (**a**) and specificity (**b**) values that are significantly different.

**Figure 3:**
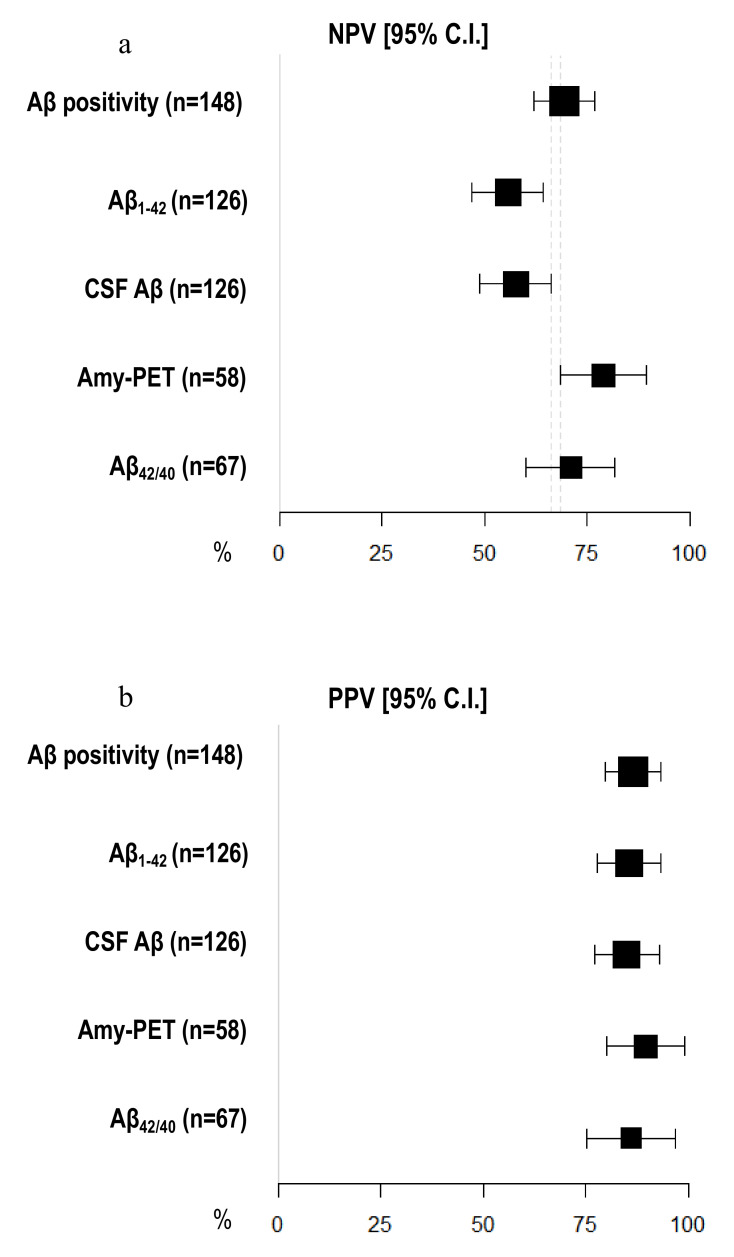
**Comparison of negative and positive predictive values among different Aβ biomarkers.** (**a**) *X*-axis represents negative predictive value (NPV) in % (95% C.I.); different amyloid biomarkers are shown in *Y*-axis. (**b**) *X*-axis represents positive predictive value (PPV) in % (95% C.I.); different amyloid biomarkers are shown in *Y*-axis. The area of each square is proportional to the degree of the distribution of values of positive and negative predictive values of each biomarker and to the number of patients for whom each biomarker is available. The whiskers represent 95% C.I. The dotted vertical lines separate NPV (**a**) and PPV (**b**) that are significantly different.

Amyloid-PET sensibility was statistically significantly higher than CSF Aβ biomarkers (89.74%, 95% C.I. 81.94–97.55 vs. 64.56%, 95% C.I. 56.20–72.91) and CSF Aβ_1–42_ (60.76%, 95% C.I. 52.23–69.29%), but not the Aβ_42/40_ ratio (77.50%, 95% C.I. 67.50–87.50%). We did not detect any differences in specificity between amyloid-PET and CSF Aβ biomarkers (Figure 2) (Table 2). Moreover, the NPV of amyloid-PET (78.95%, 95% C.I. 68.46–89.44) was statistically significantly higher than the NPVs of CSF Aβ_1–42_ (55.71%, 95% C.I. 47.04–64.39) and CSF Aβ (57.58%, 95% C.I. 48.95–66.21) (Figure 3) (Table 3).

## 4. Discussion

One aim of this study was to evaluate the diagnostic accuracy, sensitivity, specificity, and positive and negative predictive values of single or combined Aβ biomarkers. We found that the concordance with clinical diagnosis, sensitivity, and NPV of amyloid-PET were higher than CSF Aβ_1–42_ but not the CSF Aβ_42/40_ ratio. In more detail, the sensitivity and specificity of the amyloid-PET scan were 90% and 79%, respectively. Considering the possible overlap of clinical symptoms between AD and other types of dementia [43], amyloid-PET can be used to discriminate the different forms of dementia. Rabinovici et al. reported a sensitivity of 89% and a specificity of 83% of amyloid-PET in differentiating AD and FTD [44]. Previous studies showed similar diagnostic accuracy of CSF Aβ_1–42_ and florbetapir amyloid-PET [45,46], with a slightly higher diagnostic specificity for PET reported by Mattson et al. [47].

Previous studies have shown that CSF Aβ_1–42_ alone is relatively poor in distinguishing AD from other neurodegenerative disorders [25] and our results are in line with previous data. Interestingly, our results also showed that the diagnostic accuracy, sensitivity, and NPV of CSF Aβ biomarkers were significantly improved when the Aβ_42/40_ ratio was analyzed, as reported by several previous works [25,48,49,50]. Moreover, when concordance between CSF Aβ_1–42_ and Aβ_42/40_ ratio was evaluated, we found only three AD discordant patients, and all of them had a positive Aβ_42/40_ ratio but negative Aβ_1–42_. In fact, CSF Aβ_1–42_ measurements are influenced by several factors, thus false negatives may be found, leading to misinterpretation of the CSF profile. Aβ_40_ is the most abundant soluble Aβ peptide and less likely than Aβ_1–42_ to aggregate; thus, the use of a ratio may account for interindividual physiological differences in amyloid processing [50]. Therefore, recent recommendations suggested that the Aβ_42/40_ ratio should always be analyzed, irrespective of the results of other AD biomarkers [51]. Our results further support these conclusions.

Another aim of our study was to compare the concordance between the amyloid-PET and CSF Aβ biomarkers in the group of patients who underwent both the analyses. Regarding AD patients, we found a very low concordance (39%), which was different from previous works showing higher levels of concordance: Leuzy et al. reported a concordance around 60% [52], Jung et al. of 85% [53], de Wilde et al. and Reimand et al. of 91% [54,55]. Several alternative mechanisms have been proposed to explain discordance between amyloid-PET and CSF Aβ biomarkers. Previous studies showed that the majority of discordant cases were CSF+/PET−, with the highest proportion of CSF+/PET− profiles observed in cognitively normal individuals [56,57,58]. It was hypothesized that CSF biomarkers are able to detect Aβ accumulation earlier than amyloid-PET [58,59,60]. However, it is not exactly known which biomarker becomes positive first. CSF Aβ_1–42_ may also be affected by variations in amyloid precursor protein (APP) processing, Aβ production [61,62,63], and non-fibrillar aggregation [64]. Moreover, it could be reduced in other medical conditions where plaques are not present [65,66]. Finally, it could be influenced by analytical artifacts [67]. On the other hand, amyloid-PET may yield false positive results with the increasing age of patients with non-Aβ [68]. False-negative PET scans may reflect reduced sensitivity to the detection of advanced amyloid pathology, possibly caused by distinct conformations of amyloid plaques with atypical forms of Aβ pathology [69], amyloid deposition in reference regions, or severe neurodegeneration [68]. Moreover, despite current research inferring that CSF analysis and amyloid-PET could be interchangeably used in the clinic, it is well known that these two methods gauge somewhat different aspects of AD pathophysiology: CSF Aβ_1–42_ measures soluble forms of Aβ, and a low concentration suggests that significant parenchymal deposition has occurred, whereas amyloid imaging directly identifies fibrillar Aβ in brain tissue [3,69].

Despite previous works describing a higher prevalence of CSF+/PET- discordant cases compared to CSF-/PET+ [56,57,58], our findings showed a higher prevalence of patients with positive amyloid-PET but with negative CSF Aβ biomarkers. In fact, in our subgroup of AD patients who underwent both biomarkers analyses, only one patient was CSF+/PET-. In this case, CSF analysis was performed 2 months before amyloid-PET. On the other hand, 13 AD patients were CSF-PET+: the majority of these cases underwent CSF analysis more than 3 months before amyloid-PET, so we can hypothesize that this discordance could be explained by this temporal distance. Moreover, most of these patients presented CSF Aβ levels, both of Aβ_1–42_ and of the Aβ_42/40_ ratio (when available), slightly higher than cut-off values. These data are in accordance with previous reports, which suggested that amyloid-PET scans could have clinical utility in addition to CSF examination, where results are borderline and diagnostic uncertainty remains [70].

Nevertheless, our results showed that none of the patients with a clinical diagnosis of AD presented both negative Aβ biomarkers, in accordance with previous works showing that no subjects diagnosed with AD during life had a non-AD pathological diagnosis [25].

In our cohort, concordance between CSF Aβ biomarkers and amyloid-PET was higher in FTD patients than in AD patients, since the two biomarkers were both negative in 70% of cases. According to a multicenter European memory clinic study [56], the concordance rate in subjects diagnosed with FTD was 55%, lower than what we reported in our subgroup. However, 30% of our patients presented at least one positive amyloid biomarker. In more detail, two patients with clinical diagnosis of FTD were CSF+/PET-, one patient was CSF-/PET+, and only one FTD patient presented both CSF Aβ+ and positive amyloid-PET (CSF+/PET+). Amyloid positivity could be age-related, and the cognitive impairment might not be due to the amyloid burden shown by biomarkers. In fact, positive Aβ biomarkers in non-AD syndromes do not necessarily mandate a diagnostic change, because Aβ could be considered comorbid to a primary pathology that drives the clinical presentation [54]. A previous work reported that Aβ biomarkers’ positivity had been detected in 20 to 30% of cognitively normal individuals and also that patients with non-AD dementias (such as FTD) could present positive amyloid-PET, especially elderly cases carrying an APOE ε4 allele [68].

Interestingly, we found a trend toward significance for the ApoE ε4 allele in FTD patients with Aβ positivity. It has been proposed that the presence of an ApoE ε4 allele could increase AD co-pathology across neurodegenerative diseases [71].

In the FTD group in which both amyloid biomarker analyses were performed, all Aβ+ cases had a clinical diagnosis of sv-PPA. It is well known that PPA is a group of neurodegenerative diseases with heterogeneous neuropathologic causes. A recent work showed that Aβ positivity was detected in 25% of sv-PPA cases and in 40% of patients with mixed PPA with overlapping linguistic features for sv-PPA and lv-PPA (s/lv-PPA) [42]. A recent meta-analysis on 1251 PPA patients reported that Aβ positivity was found in 16% of sv-PPA [72]. Therefore, positive amyloid biomarkers in our patients with FTD clinical diagnosis could also be linked to the inclusion of patients with sv-PPA.

**Figure 4:**
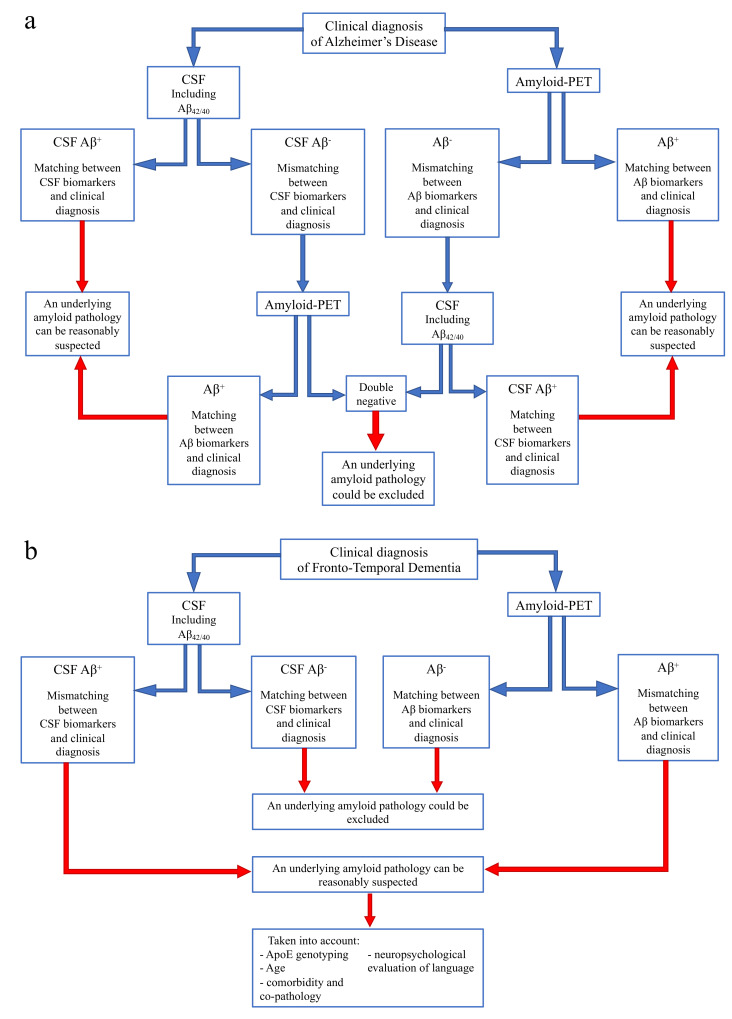
**Flow chart for use and interpretation of amyloid biomarkers according to clinical suspect.** (**a**) Indication for interpretation of amyloid biomarkers in the case of a clinical diagnosis of Alzheimer’s disease. (**b**) Indication for interpretation of amyloid biomarkers in the case of a clinical diagnosis of Frontotemporal dementia.

On the basis of our current and previous data [73], we would like to suggest a flowchart to guide the use and interpretation of amyloid biomarkers according to different clinical diagnoses, in order to reach conclusions in specific clinical situations (Figure 4).

1. In case of a clinical diagnosis of AD [16] (Figure 4a):
  - If CSF analysis including Aβ_42/40_ ratio is positive, underlying Aβ pathology can be reasonably suspected and amyloid-PET might be avoided. Similarly, if amyloid-PET detects cortical amyloid deposition, CSF analysis cannot be performed. In conclusion, as also reported in a recent study [73], the matching between clinical diagnosis and a single amyloid biomarker could be sufficient, considering the high PPV of both CSF analysis and amyloid-PET.
  - In case of a mismatch between clinical diagnosis and one amyloid biomarker, we suggest performing the other analysis, due to the low NPV of both CSF and amyloid-PET. In particular:
    ○ If CSF shows Aβ-, amyloid-PET should be performed, also considering previous results highlighting an advantage of amyloid-PET when used as a second biomarker [74].
    ○ If amyloid-PET is negative for cortical deposition, CSF analysis including Aβ_42/40_ ratio is suggested.
  In conclusion, if both the analyses show Aβ-, an underlying amyloid pathology could most probably be excluded, and a revision of clinical diagnosis should be considered. On the contrary, if the second analysis detects Aβ+, and matching between clinical diagnosis and biomarkers is achieved, amyloid pathology can be reasonably suspected.
2. In case of a clinical diagnosis of FTD [26] (Figure 4b):
  - If CSF analysis or amyloid-PET shows Aβ-, an underlying amyloid pathology could most probably be excluded. Thus, the performance of the other biomarker is not required, due to the high concordance between these two analyses in FTD cases.
  - If CSF or amyloid PET detects Aβ+, some factors should be taken into account, in particular ApoE genotyping, age, and a deep neuropsychological evaluation of language to exclude a diagnosis of PPA. Moreover, the presence of comorbidity and co-pathology should be considered, as also suggested by other recent reports [73].

Several studies have highlighted that biomarkers strongly improved the diagnostic accuracy of clinical diagnostic criteria, and it has been suggested that their combination seems to add accuracy to clinical evaluation [75]. However, their use presents some limitations due to the cost of amyloid-PET and the invasiveness of CSF analysis, especially in primary care settings. In the future, the use of blood biomarkers could be validated, in addition to an accurate clinical and neuropsychological diagnosis, due to their potential low cost and low invasiveness. Current research is focusing on these promising biomarkers, although further work is needed on both clinical and analytical validation of these candidates. These blood biomarkers might be helpful not only in early identification of neurodegeneration but also as a starting point to differentiate AD from FTD.

The main limitation of our study was the lack of autopsy confirmation of Aβ pathology. We only tested the associations between amyloid biomarkers and clinical diagnosis and the concordance between CSF Aβ biomarkers and amyloid-PET, and it is possible that some subjects were clinically misdiagnosed with AD. However, we detected GRN pathogenetic mutations in two cases with nfv-PPA, so we can diagnose these patients with PPA with defined pathology [76]. Secondly, the retrospective design of the study could have led to some bias: not all patients had both biomarkers analyzed as CSF analysis of the Aβ_42/40_ ratio was not available in all cases. Moreover, the subgroup of patients who underwent both the analyses was relatively small, making it difficult to perform multivariate analysis to correct for possible confounding factors. Furthermore, CSF and amyloid-PET were not performed at the same time. Another limitation was that FTD patients who underwent both amyloid biomarkers had amyloid-PET performed before CSF analysis, so we cannot exclude that differences in the proportion of Aβ positivity between AD and FTD were influenced by the time between the two analyses. Moreover, APOE genotyping was not available for all patients.

However, our study presents some remarkable strengths. First of all, a relatively large number of patients of our cohort underwent at least one amyloid burden biomarker. Another strength of this study is that it was based on a clinical setting cohort; therefore, it provides realistic information on the use and interpretation of amyloid biomarkers. Third, all patients were evaluated by an extensive neuropsychological battery and their clinical diagnosis was well defined.

## 5. Conclusions

In conclusion, due to the high PPV of both amyloid-PET and CSF analysis including Aβ_42/40_, in cases of concordance between at least one amyloid biomarker and clinical diagnosis, performance of the other analysis could be avoided; on the other hand, a combination of both amyloid-PET and CSF biomarkers should be performed to better characterize unclear cases of dementia. If the two biomarkers of amyloid burden are both negative, an underlying AD pathology could most probably be excluded.

## Figures and Tables

**Table 1:** Demographic variables, cognitive data, and ApoE ε 4 proportion.

Demographic	AD (*n* = 95)	FTD (*n* = 53)	*p*
Age at baseline (±SD)	66.69 (±7.98)	67.04 (±8.17)	0.650
Age at onset (±SD)	63.49 (±8.88)	64.24 (±8.27)	0.661
Age at CSF analysis (±SD)	68.02 (±8.04)	67.86 (±7.50)	0.906
Age at amyloid-PET (±SD)	66.76 (±7.34)	67.36 (±8.73)	0.587
Time from CSF to amyloid-PET (±SD) *	0.562 (±0.78)	−0.18 (±0.88)	**0.034**
Disease duration (±SD)	3.03 (±3.31)	2.61 (±1.84)	0.951
Sex (women/men)	54/41	28/25	0.638
Familiality (%)	41.40	50.00	0.902
Education (±SD)	10.56 (±5.10)	9.37 (±4.20)	0.205
TIB (±SD)	108.70 (±8.02)	104 (±4.03)	0.292
MMSE (±SD)	19.55 (±5.44)	20.32 (±5.68)	0.400
HDRS (±SD)	28.45 (±5.61)	30.75 (±6.18)	0.525
ApoE ε4+ (%)	24.48	3.69	**0.008**

Values quoted in the table are mean (±SD). Age at baseline, age at onset, age at CSF analysis, age at amyloid-PET, disease duration, and schooling are expressed in years. Age at baseline indicates age at the baseline evaluation; age at onset indicates age at the onset of symptoms; disease duration indicates time from onset of symptoms and baseline evaluation; follow-up time indicates the time from baseline visit to the last evaluation. * indicates time from CSF analysis and amyloid-PET, and it refers only to patients who underwent both amyloid biomarkers analyses. *p* indicates level of significance for comparison between AD and FTD. TIB (*Test di Intelligenza Breve*); HDRS (Hamilton Depression Rating Scale). The bold: statistically significatant results.

**Table 2:** Concordance between CSF biomarkers and amyloid-PET and between CSF Aβ_1–42_ and Aβ_42/40._

		Amyloid-PET +	Concordance [95% C.I.] %
**CSF Aβ+**	AD	9/23 (39.13%)	39.13% [19.94–63.40]
	FTD	1/12 (8.33%)	76.92% [54.02–99.83]
	Total	10/36 (27.28%)	52.78% [36.47–69.09]
		**Aβ_42/40_ +**	**Concordance [95% C.I.] %**
**Aβ_1–42_ +**	AD	28/40 (70.00%)	92.50% [84.34–100]
	FTD	4/27 (7.41%)	85.19% [71.79–98.59]
	Total	32/67 (47.76%)	89.55% [82.23–96.88]

CSF Aβ was defined as positive if at least one Aβ_1–42_ level or Aβ_42/40_ ratio was reduced under cut-off values. Aβ_1–42_ cut-off: ELISA = 600.00 pg/mL, LUMIPULSE = 670.00 pg/mL; Aβ_42/40_ cut-off = 0.062.

**Table 3:** Sensitivity, specificity, and accuracy of each biomarker.

	Sensitivity [95% C.I.]	Specificity [95% C.I.]	Accuracy [95% C.I.]
	**(%)**	**(%)**	**(%)**
Aβ positivity (*n* = 148)	81.05 [74.74–87.37]	77.36 [70.62–84.10]	79.73 [73.25–86.21]
CSF Aβ (*n* = 126)	64.56 [56.20–72.91]	80.85 [73.98–87.72]	70.63 [62.68–78.59]
Aβ_1–42_ (*n* = 126)	60.76 [52.23–69.29]	82.98 [76.42–89.54]	69.05 [60.98–77.12]
Aβ_42/40_ (*n* = 67)	77.50 [67.50–87.50]	81.48 [72.18–90.78]	79.10 [69.37–88.84]
Amyloid-PET (n = 58)	89.74 [81.94–97.55]	78.95 [68.46–89.44]	86.21 [77.33–95.08]

Aβ positivity was defined if at least one of the amyloid biomarkers (CSF or amyloid PET) revealed the presence of Aβ pathology. CSF Aβ was defined as positive if at least one of either the Aβ_1–42_ level or the Aβ_42/40_ ratio was reduced under cut-off values. Aβ_1–42_ cut-off: ELISA = 600.00 pg/mL, LUMIPULSE = 670.00 pg/mL; Aβ_42/40_: cut-off = 0.062.

## Data Availability

Raw data that support the findings of this study are not presented due to ethical and patient data security reasons but will be shared upon request from any qualified investigator.
